# The effect of icariin for infertile women with thin endometrium: A protocol for systematic review

**DOI:** 10.1097/MD.0000000000019111

**Published:** 2020-03-20

**Authors:** Juan Du, Hua Lu, Xujun Yu, Liang Dong, Ling Mi, Jinpeng Wang, Xia Zheng, Kai Feng

**Affiliations:** a Hospital of Chengdu University of Traditional Chinese Medicine; b Chengdu University of Traditional Chinese Medicine; c The Reproductive & Women-Children Hospital, Chengdu University of Traditional Chinese Medicine, Chengdu, Sichuan, P.R. China.

**Keywords:** icariin, infertility, meta-analysis, protocol, systematic review, thin endometrium

## Abstract

**Background:**

Thin endometrium, defined as <7 mm of the endometrial thickness around ovulation period, had been identified as a negative factor on pregnancy rate of infertile women. It was considered to be the toughest part in treatment of infertility, because there was a lack of significant effect, although many drugs had been already used. Icariin was one of the major bioactive pharmaceutical constituent extracted from the Chinese herb “Ying Yang Huo,” in the genus of Epimedium, and some randomized controlled trials reported its application for thin endometrium. There is no systematic review focusing on the effective of icariin in treating infertile women with thin endometrium, so our review aims to explore it.

**Methods:**

The bibliographic database and electronic library will be systematically searched online, such as MEDLINE, EMBASE, Web of Science, Clinicaltrails.org., China National Knowledge Infrastructure Database (CNKI), Wan fang Database, China Biology Medicine Database (CBM), VIP Science Technology Periodical Database, and Cochrane Library. And the reference listed for potential literatures of included studies will be scanned additionally. Related randomized controlled trials (RCTs) will be collected and selected before January 4, 2020. Trials will be screened by independent reviewers, and the literature will be search in English or Chinese, with the search terms as “Icariin,” “Epimedium,” “infertile women,” “female infertility,” “endometrium,” “pregnancy rate.” The software for Systematic review and Meta-analysis is RevMan 5.3. The protocol and the systematic review will be reported according to the Preferred Reporting Items for Systematic Reviews and Meta-Analyses Protocols (PRISMA-P) statement.

**Result and conclusion:**

The efficacy of icariin to treat thin endometrium will be evaluated, and the conclusion will be published to help clinicians determine treatment strategy for infertile women with thin endometirum by providing medical evidence.

**Registration information:**

PROSPERO CRD42019148977.

## 1 Introduction

Infertile women are female patients who suffer from the clinical pregnancy failure after 12 months regular sexual intercourse without conception. The morbidity is between 8% and 12% in reproductive-aged couples worldwide.^[1]^ The global disease burden of infertility had been increasing throughout the period from 1990 to 2017.^[2]^ So reproductive medicine has received widespread attention and become a research highlights. The primary assessments for infertile women are the ovulation, the uterine cavity, and the fallopian tubes.^[3]^

As an important part of uterine factors for female infertility, endometrial thickness is one of the most fundamental conditions for embryo implantation. Numerous studies showed that there were significant differences of pregnancy rate between thin endometrium patients and normal ones, so thin endometrium had been regarded as an independent negative factors for pregnancy, whether in assisted reproductive technology (ART) or in natural pregnancy.^[4–10]^

What is defined as “thin endometrium” which interferes with the implantation of embryos? The existed studies were almost based on the researches in ART, but the experiences in diagnosis were derived to other infertile women with thin endometrium. Thin endometrium is considered that the endometrial thickness is <7 mm on the ovulatory day, or on the human chorionic gonadotropin (HCG) injecting day in controlled ovarian hyperstimulation (COH) cycles, or the day to start using progesterone in frozen-thaw embryo transfer cycles, according to the guideline of the Canadin Fertility and Andrology Society, and the Chinese expert consensus of the Reproductive Medicine Society in Chinese Medical Association.^[11,12]^ And the prevalence of this disease was reported as 2.4% in the ART.^[13]^ “Thin endometrium” was still supposed to be the toughest part of infertility treatment, which was lack of significant effect, although many drugs had been already used for treatment.

Icariin is one of the major bioactive pharmaceutical constituent extracted from the Chinese herb named “Ying Yang Huo” or Horny Goat Weed, in the genus of Epimedium, commonly used as a tonic, aphrodisiac, anti-rheumatic, and anti-cancer agent in traditional herbal formulations in Asian countries like China, Japan, and Korea.^[14,15]^ Icariin was proved to promote the proliferation of the cells and the repairment of tissues. It was confirmed by animal experiments that icariin had potentiality to accelerate cutaneous wound healing in rats.^[16]^ As a result, icariin had been used in many diseases, such as osteoporosis,^[17–19]^ steroid-associated osteonecrosis,^[20]^ osteochondral injuries,^[21,22]^ and so on, and the freeze-dried platelet-rich plasma carrying icariin could provide sustained release of icariin into the tendon-bone healing site, thus effectively accelerating tendon-bone healing as icariin providing strong stimulation for osteogenesis.^[23]^

Icariin had the function of neuroprotection by the promotion roles in cell proliferation and the regulatory roles in gene expression in human neural stem cells (NSCs) in vitro to regulate NSC activity,^[24]^ and the neuroprotective properties were used against brain dysfunction for Parkinson disease,^[25]^ spinal cord injury,^[26]^ and even cardiac remodeling following myocardial infarction.^[27]^ And icariin acted as a nerve growth factor-releasing agent, and it indicated that local application of icariin after spinal injury could promote peripheral nerve regeneration.^[28]^

A review on the effect of plant extract on mesenchymal stem cell proliferation and differentiation showed certain bioactive compounds from phytochemicals like icariin increased the rate of tissue regeneration, differentiation, and immunomodulation, by a specific role (bioactive mediator) in regulating the rate of cell division and differentiation through complex signal pathways.^[29]^ Icariin promoted the migration and proliferation of keratinocytes, and accelerated the healing of skin wounds via the up-regulation of AKT serine/threonine kinase 1(AKT) and extracellular signal-regulated kinase (ERK) signaling.^[30]^ Another research reported icariin had shown great potential in improving cell activity and vascular endothelial growth factor (VEGF), brain-derived neurotrophic factor (BDNF) secretion in vitro, and icariin and mesenchymal stem cells synergistically promoted angiogenesis and neurogenesis after cerebral ischemia via Phosphotylinosital 3 kinase (PI3K) and extracellular regulated protein kinases1/2(ERK1/2) pathways.^[31]^

In the theory of traditional Chinese medicine (TCM), the efficacy of epimedium, the source of icariin extraction, is tonifying kidney-yang. It was proved that icariin might be therapeutically beneficial in the treatment of kidney yang syndrome, through attuning the hypothalamus-pituitary-adrenal (HPA) axis and endocrine system which was involved in the release of corticotropin releasing hormone (CRH) in hypothalamic, and the production of pituitary proopiomelanocortin (POMC) derived peptide adrenocorticotropic hormone (ACTH) in anterior pituitary, the secretion of corticosteroids in adrenal cortex.^[32]^

An experimental study on rats demonstrated that icariin increases thickness of the endometrium, and it may modulate expression of VEGF, Cluster of Differentiation 31(CD31), and factor VIII, which were primarily expressed in the cytoplasm of endometrial and vascular epithelial cells.^[33]^ Some trials reported the application of icariin treating thin endometrium of women infertility, and showed its effectiveness. So our reviewer team focused on the effect of icariin for infertile women with thin endometrium.

### 1.1 Review objectives

This review is aimed to retrieve, identify, and assess all studies to evaluate the efficacy of icariin for infertile women with thin endometrium, comparing with other treatment groups, placebo groups, or untreated control group. We will establish the differential efficacy of interventions for thin endometrium in consideration of endometrial thickness, pregnancy rate, to provide medical evidence for making better treatment strategy for thin endometirum in women infertility.

## 2 Methods

As a systematic review, all the information will be extracted from the previously published studies, ethical approval is not necessary.

### 2.1 Protocol and registration

This protocol will be reported in line with the Preferred Reporting Items for Systematic Reviews and Meta-Analyses Protocols (PRISMA-P) statement.^[34]^ This review has been registered on PROSPERO international prospective register of systematic reviews (www.crd.york.ac.uk/prospero), with the registration number: CRD42019148977.

### 2.2 Data source

#### 2.2.1 Electronic search database and approach

The bibliographic database and electronic library will be systematically searched online, such as MEDLINE, EMBASE, Web of Science, Clinicaltrails.org., China National Knowledge Infrastructure Database (CNKI), Wan fang Database, China Biology Medicine Database (CBM), VIP Science Technology Periodical Database, and Cochrane Library. Related randomized controlled trials (RCTs) will be collected and selected before January 4, 2020. Trials will be screened by independent reviewers, and the literature will be search in English or Chinese, with the search terms as “Icariin,” “Epimedium,” “infertile women,” “female infertility,” “endometrium,” “pregnancy rate,” and these key words will be translated into Chinese for searching in Chinese database. It is necessary to apply the corresponding retrieval strategies in different databases, and here is the search example for MEDLINE in Table 1.

**Table 1:**
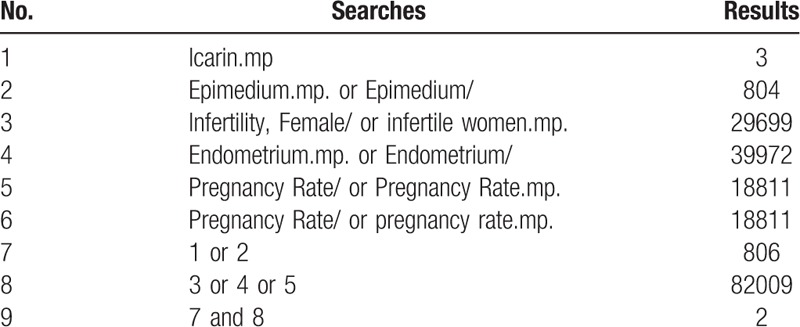
MEDLINE search strategies.

#### 2.2.2 Other sources of search

OpenGrey will be searched for data and information in grey literature. The whole document of articles will be got through Internet or electronic libraries. The reference listed for potential literatures of included studies will be manually scanned. For the original detail of related conference papers or dissertations, the authors will be contacted.

### 2.3 Included and excluded criteria

#### 2.3.1 Study design

Only randomized controlled trials will be included, while other studies, as observational studies, non-comparative studies, self-controlled trials, case reports/series, review papers, animal experiments, or articles written in other languages except English and Chinese will be exclude.

#### 2.3.2 Participants

Infertile women who meet the diagnostic criteria of thin endometrium mentioned above will be included, while the patients with congenital anomalies of uterine or endometrium, or severe endometrial injury, or other endometrial abnormalities except for thin endometrium will be excluded.

#### 2.3.3 Interventions

The interventions of thin endometrium include icariin or the effective chemical constituents in epimedium. Randomized, double-blind, placebo-controlled trials would be identified the best. And the control group should be another treatment group with one of estrogen, gonadotropins, platelet rich plasma, letrozole, granulocyte colony-stimulating factor, sildenafil citrate, pentoxifyllin, tocopherol, and tamoxifen, or placebo-controlled group with placebo in the same pattern, or no treatment group.

#### 2.3.4 Outcomes

##### 2.3.4.1 Primary outcome indicator

Primary outcome indicators will be consisted of endometrial thickness and pregnancy rate, which were based on the results reported at the end of included studies.

(1) Pregnancy rate: Pregnancy rate was defined as the proportion of successfully pregnant women after treatment for infertile women with thin endometrium. “Pregnancy” may refer to a positive pregnancy test, whether by blood or urine test, or B ultra-sound radiography.
(2) Endometrial thickness: Endometrial thickness was measured by B ultra-sound radiography, identified as the maximum dimension between the 2 endometrial–myometrial junctions, in the uterine midsagittal plane.^[7]^

##### 2.3.4.2 Secondary outcome indicators

Secondary outcome indicators will be consisted of miscarriage rate, endometrial pattern.

(1) Miscarriage rate: Miscarriage rate was considered as the proportion of spontaneous pregnancy loss in pregnant women.
(2) Endometrial pattern: Endometrial pattern were classified as 3 types, pattern A (triple-line type which is characterized by a hypoechoic endometrium with well-defined hyper-echoic outer walls and a central echogenic line), pattern B (isoechoic endometrium with poorly defined outer walls and central echogenic line), and pattern C (homogeneous hyper-echoic endometrium), according to the morphology of the endometrium by B ultra-sound radiography.^[35]^
(3) Adverse events.

### 2.4 Selection of studies and data extraction

**Figure 1:**
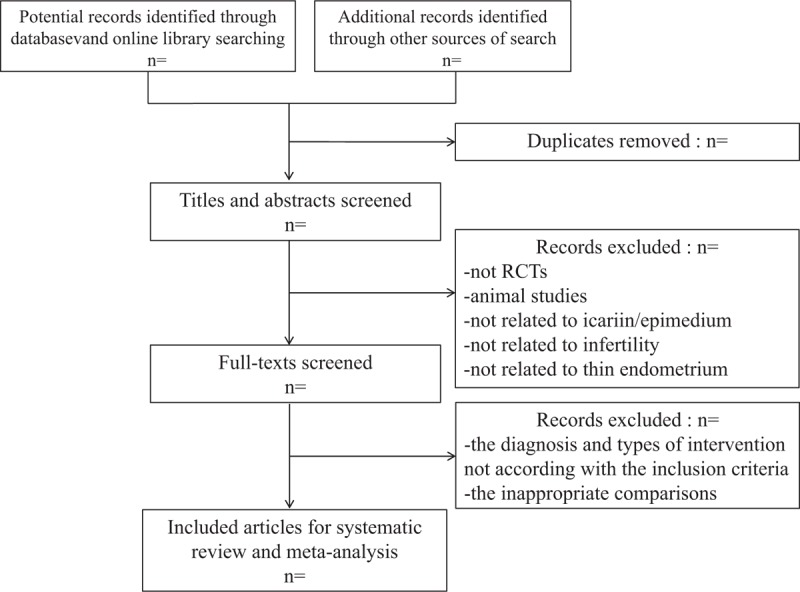
The PRISMA literature screening flow chart. PRISMA = Preferred Reporting Items for Systematic Reviews and Meta-Analyses.

Two skillful reviewers (JD, XY) will make retrieval strategies separately and all the document is managed by the software Endnote X9 (Boston, USA). After filtering and removing duplicates, all articles which comparing icariin with placebo-controlled group, or other treatment group, or no treatment group will be scanned and included. The full-text of right articles will be searched online or in electronic library, while the relative conference papers or dissertations can be obtained by contacting the corresponding author for original details. Then, after discussion in review group, a unified data extraction form (an excel spreadsheet) will be produced. Extracted information will include characteristics and methodology of included studies, participant characteristics of included studies, details of interventions and control measures, data information for outcome indicators of included studies, and other data including information for assessment of the risk of bias (Fig. 1). Before the formal data extraction, 2 review authors (JD, LD) will independently conduct data extraction exercises. All the uncertain problems will be discussed with the third reviewer (HL) to resolve.

### 2.5 Risk of bias assessment

The Cochrane Collaboration Network Risk Assessment Tool will be applied to evaluate the biases, as selection bias, performance bias, detection bias, attrition bias, reporting bias, and others. Two well-trained review authors will independently evaluate and check the risks of bias. Discrepancies between review authors on the risk of bias will be resolved through discussion with a third review author. After that, the software Revman 5.3 (London, United Kingdom) will be utilized to risk of draw the bias assessment chart.

### 2.6 Data analysis and synthesis

Descriptive analysis will be performed when there is clinical heterogeneity between the studies or when the data can not be synthesized. Meta-analysis (RevMan 5.3 software) will be used when the studies are homogeneous and the data are similar and synthesizable. Dichotomous variable will be pooled as relative risk (RR) and 95% confidence intervals (CIs). Continuous variable will be pooled as mean difference (MD) and 95% CI. We will use Cochran *Q* statistic and *I*^2^ statistic to test the heterogeneity. *P* < .10 is heterogeneous, and *I*^2^ > 50% is significant heterogeneity. A fixed effect model (Mantel-Haenzel method for RR and Inverse Variance for MD) will be used for *I*^2^ < 50%. A random effects model (D-L method) will be used when the heterogeneity is still significant after sensitivity analysis and subgroup analysis. A *P* < .05 of *Z* test will be considered statistically significant. Sensitivity analysis will be used to test the stability of the results. Publication bias will be measured by using a funnel plot (RevMan 5.3 software) or Egger test (Stata software [Texas, USA]).

### 2.7 Subgroup analysis

If the data are sufficient and there is heterogeneity between studies, we will perform a subgroup analysis. Subgroup analysis was performed according to different age, different ethnicity, different kind of male infertility, different comorbidity, different interventions, different control measures, different measurement methods, and time of measurement.

### 2.8 Sensitivity analysis

Sensitivity analysis refers to observation of the changes of heterogeneity and synthesis along with the changes of important factors which will affect the results, such as the inclusion criteria, risk of bias, loss to follow-up, statistical methods (fixed effect model, FEM or random effect model, REM) and the selection of effect size (odds ratio, OR or RR), so it's an indicator to judge the stability and reliability of the meta-analysis results.^[36]^

### 2.9 Publication bias

If the researchers fail to publish the research results, especially the negative results, the quality of evidence will be weakened. Publication bias leads to inflated treatment effect which will lower the reliability of evidence. Published bias are evaluated by Begg test and Egger test by Stata software 14.0, or a funnel plot with the software RevMan 5.3.^[37]^

## 3 Discussion

Endometrium thickness plays an important role in embryos implantation. Thin endometrium of infertile women is a difficult issue in clinical treatment. Many medicines are used to improve thin endometrium, so does icariin.

What is the possible mechanism of icariin for treating thin endometrium? It is inferred that the effects of icariin is related to its promotion of blood vessels and nerves, or its regulation to estrogen related receptors or signaling pathways. Some studies found the endometrium may be thickened by icariin treatment by increasing the expression levels of estrogen receptor (ER), VEGF, and kinase insert domain receptor (KDR) in the endometrial cells of the thin endometrium.^[38]^ And the active components of icariin included epimedium, which can be used to synthesize different prenylated flavonols, was related to estrogen signaling pathway,^[39]^ and icariin was predicted to act as ER modulator.^[40]^ In addition, icariin significantly promoted cell migration and capillary tube formation, and favorable modulation of the angiogenesis and redox states in bone marrow-derived endothelial progenitor cells (BM-EPCs) make icariin a promising proangiogenic agent both enhancing vasculogenesis and protecting against endothelial dysfunction.^[41]^ Besides, icariin acts as a nerve growth factor-releasing agent, and promotes peripheral nerve regeneration.^[28]^

This systematic review shows the limitations that the quantity of RCTs included is far from enough, maybe because of the newly application of icariin for treating thin endometrium of infertile women and the literature retrieval in English and Chinese except for other languages. More reports on relevant researches are expected.

## Author contributions

**Conceptualization:** Hua Lu, Liang Dong.

**Data curation:** Hua Lu, Xujun Yu.

**Data management:** Juan Du, Xujun Yu, Hua Lu

**Draft writing:** Juan Du, Liang Dong, Xia Zheng,Kai Feng

**Formal analysis:** Hua Lu, Xujun Yu.

**Funding acquisition:** Hua Lu.

**Methodology:** Hua Lu, Xujun Yu.

**Program management:** Juan Du, Hua Lu, Xujun Yu

**Resources:** Hua Lu.

**Research design and concept**: Juan Du, Hua Lu, Liang Dong

**Software:** Liang Dong, Jinpeng Wang, Xia Zheng.

**Writing – original draft:** Liang Dong, Jinpeng Wang, Xia Zheng, Kai Feng.

**Writing – review & editing:** Hua Lu, Liang Dong, Ling Mi.
